# Musculoskeletal Telemedicine Trends Preceding the COVID-19 Pandemic and Potential Implications of Rapid Telemedicine Expansion

**DOI:** 10.1155/2023/9900145

**Published:** 2023-01-11

**Authors:** Sara N. Kiani, Logan D. Cho, Jashvant Poeran, Lauren Wilson, Haoyan Zhong, Madhu Mazumdar, Jiabin Liu, Alejandro Gonzalez Della Valle, Stavros G. Memtsoudis

**Affiliations:** ^1^Department of Medical Education, Icahn School of Medicine at Mount Sinai, New York, NY, USA; ^2^Institute for Healthcare Delivery Science, Department of Population Health Science and Policy, USA; ^3^Department of Orthopedics, Icahn School of Medicine at Mount Sinai, One Gustave L. Levy Place, New York, NY, USA; ^4^Department of Anesthesiology, Critical Care & Pain Management, Hospital for Special Surgery, New York, NY, USA; ^5^Department of Anesthesiology, Weill Cornell Medical College, New York, NY, USA; ^6^Department of Orthopedics, Hospital for Special Surgery, New York, NY, USA; ^7^Department of Healthcare Policy and Research, Weill Cornell Medical College, New York, NY, USA; ^8^Department of Anesthesiology, Perioperative Medicine and Intensive Care Medicine, Paracelsus Medical University, Salzburg, Austria

## Abstract

**Introduction:**

Telemedicine was rapidly deployed at the onset of the COVID-19 pandemic. Little has been published on telemedicine in musculoskeletal care prior to the COVID-19 pandemic. This study is aimed at characterizing trends in telemedicine for musculoskeletal care preceding the COVID-19 pandemic.

**Methods:**

This retrospective study used insurance claims from the Truven MarketScan database. Musculoskeletal-specific outpatient visits from 2014 to 2018 were identified using the musculoskeletal major diagnostic category ICD-10 codes. Telemedicine visits were categorized using CPT codes and Healthcare Common Procedure Coding Systems. We described annual trends in telemedicine in the overall dataset and by diagnosis grouping. Multivariable logistic regression modeling estimated the association between patient-specific and telemedicine visit variables and telemedicine utilization.

**Results:**

There were 36,672 musculoskeletal-specific telemedicine visits identified (0.020% of all musculoskeletal visits). Overall, telemedicine utilization increased over the study period (0% in 2014 to 0.05% in 2018). Orthopedic surgeons had fewer telemedicine visits than primary care providers (OR 0.57, 95% CI 0.55-0.59). The proportion of unique patients utilizing telemedicine in 2018 was higher in the south (OR 2.28, 95% CI 2.19-2.38) and west (OR 5.58, 95% CI 5.36-5.81) compared to the northeast. Those with increased comorbidities and lower incomes and living in rural areas had lower rates of telemedicine utilization.

**Conclusions:**

From 2014 to 2018, there was an increase in telemedicine utilization for musculoskeletal visits, in part due to insurance reimbursement and telemedicine regulation. Despite this increase, the rates of telemedicine utilization are still lowest in some of the groups that could derive the most benefit from these services. Establishing this baseline is important for assessing how the roll-out of telemedicine during the pandemic impacted how/which patients and providers are utilizing telemedicine today.

## 1. Introduction

Telemedicine in musculoskeletal care offers great promise. Individual hospitals have published data showing increased telemedicine utilization since the start of the pandemic [1, 2], but this has not yet been shown on a national scale. Unfortunately, there has been limited research in the United States documenting prepandemic baseline telemedicine rates or telemedicine trends in musculoskeletal care.

Widespread implementation of telemedicine in musculoskeletal care may lead to an improvement in patient care and health outcomes. Prior analyses of telemedicine in musculoskeletal settings have shown that patient safety is not compromised with its use [3–5], and patients are comparably satisfied with the remote interactions [6–8]. In addition, these visits are cost-effective for both patients and health systems [8–10]. However, despite these advantages, patients that have lower median household income and/or live in rural areas—those who have the most to theoretically gain from telemedicine—often have been the least able to access it [11].

Restrictive reimbursement rates have limited the expansion of telemedicine services in the past [12]. Medicare, in particular, has consistently been one of the most restrictive in telemedicine coverage, and many commercial payers have also implemented similar restrictions [13]. This may in part be due to a desire to minimize costs, as individuals have higher healthcare utilization when telemedicine is available [14]. With increasing public and private reimbursement since the onset of the COVID-19 pandemic, telemedicine has expanded across all specialties, including musculoskeletal care. Many musculoskeletal and orthopedic clinics began to offer telemedicine for the first time following the onset of the pandemic [2, 15], with 83% of academic orthopedic surgery clinics offering telemedicine services as a direct result of the COVID-19 pandemic [15]. The institutions most likely to offer telemedicine were located in the northeast and south regions of the United States, regions that were “hot spots” of COVID-19 at the time of the article's publication [15]. Without baseline data on differences in telemedicine utilization, it cannot be concluded if this correlation is truly a result of infection rates, as these regional differences could have existed prepandemic.

This study is aimed at (1) documenting the prevalence of musculoskeletal telemedicine utilization within the United States, (2) analyzing trends in telemedicine visits and patients over the 4-year study period, and (3) tracking these trends by visit subtype. These prepandemic rates of telemedicine utilization in musculoskeletal care establish a baseline necessary for contextualizing telemedicine trends following the onset of the COVID-19 pandemic. This contribution allows for better analysis of the deployment of telemedicine in musculoskeletal care.

## 2. Materials and Methods

Approval of this study was obtained from the Institutional Review Board.

### 2.1. Study Design, Database, and Sample

This retrospective study used patient-level private insurance claims from >100 payers from the Truven MarketScan database to identify telemedicine visits that occurred between 2014 and 2018 (*n* = 846,461,609 visits; copyright © 2017 Truven Health Analytics Inc.; dataset access was limited to Hospital for Special Surgery employees). To extract musculoskeletal-specific outpatient visits (*n* = 190,299,246 visits), the cohort was filtered using ICD-10 codes that are associated with the musculoskeletal major diagnostic category (MDC). Telemedicine visits were defined using current procedural terminology codes 99441-99444; Healthcare Common Procedure Coding System codes G0406-G0408, G0459, G0508-G0509, G0425-G0427, Q3014, and T1014; or any code with either a procedure modifier of GT, GQ, or 95 or a location of service listed as “telehealth” [16].

### 2.2. Study Variables

The primary outcome of this analysis was the utilization of telemedicine as the modality of a patient visit. The study variables considered included both patient-specific variables and telemedicine visit characteristics.

Patient-specific variables included the following: sex, age, Charlson-Deyo Comorbidity Index (categories: 0, 1, 2, >2, with a higher score representing a higher comorbidity burden) [17], active opioid use, residence rurality (urban, rural), residence geographic region (northeast, north central, south, west, unknown), and median household income.

Telemedicine visit characteristics included the following: diagnosis, encounter type, provider type, copayment, and year of visit (2014-2018). Using ICD-10 codes, the diagnoses were classified into 5 major groups (hip/knee pain, low back or neck pain, musculoskeletal aftercare, and other). The encounter type was determined using the MarketScan variable “SVCSCAT,” which identifies the detailed service type (21225: office visits, nonspecialized physician, likely primary care; 21125: office visits, specialized physician, likely surgeon, pain physician, physiatrist, etc.; 22325, 22335, and 12335: professional OP visits, likely mostly representing PT; 21299, 12399, 21199, 12328, 22399, 20226, 12220, 22330, 21120, 22333, 45168, 22315, 20126, 2115, 21226, and 12215: other). Provider type was classified as primary care, urgent care, orthopedic surgeon, and other.

### 2.3. Statistical Analysis

In this paper, we analyzed annual trends in telemedicine for adults > 18 who had a visit classified as “MDC code 8 (musculoskeletal).” This analysis was performed both for the overall dataset and by diagnosis group. Cochran-Armitage trend tests assessed linear trends.

The multivariable logistic regression model estimated the association between both patient-specific and telemedicine visit study variables and telemedicine utilization. Odds ratios (OR) and 95% confidence intervals (CI) were reported; analyses were performed using SAS statistical software v9.4.

## 3. Results

From 2014 to 2018, a total of 190,299,246 adult outpatient visits classified with MDC code 8 (musculoskeletal) were observed in our study. Of these, 36,672 were telemedicine visits (0.020%). The number of unique telemedicine patients ranged from 1837 (0.018%) to 15,338 (0.270%) over the years of the study. The majority of people who used telemedicine had just 1 visit (*n* = 28,831). Of the remaining patients, 2885 had 2 telemedicine visits, and 1231 had over 2 visits.

**Figure 1:**
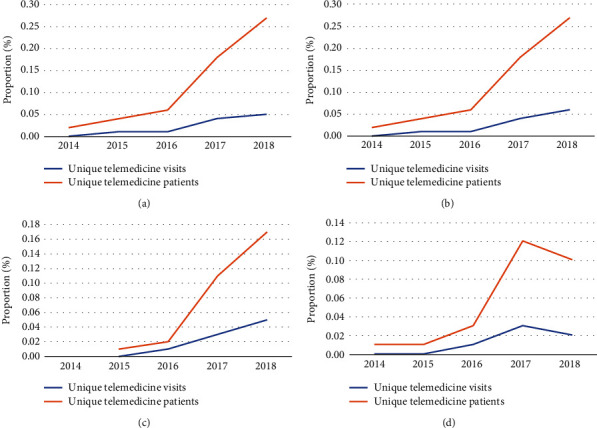
Trends in unique telemedicine visits/patients for (a) all adults (>18) with MDC code 8, (b) with only low back or neck pain, (c) with only hip/knee pain, and (d) musculoskeletal aftercare.

Telemedicine utilization increased over the study period from nearly 0% to 0.05% of visits and 0.02% to 0.27% of patients, with the proportion of telemedicine patients increasing more quickly than telemedicine visits (Figure 1(a)). An inflection point is observed in 2016, with a significant increase in the number of patients utilizing telemedicine in the subsequent years. Similar patterns were seen for low back or neck pain (0% to 0.06% of visits; 0.02% to 0.27% of patients) (Figure 1(b)) and hip/knee pain (0% of visits to 0.05% of visits; 0.01% to 0.17% of patients) (Figure 1(c)). Each year, there was an increase in telemedicine visits and patients for these groups. Musculoskeletal aftercare was unique because while there was an overall increase in visits and patients (0% to 0.02% of visits; 0.01% to 0.1% of patients), there was a decrease from 2017 to 2018 (Figure 1(d)).

**Figure 2:**
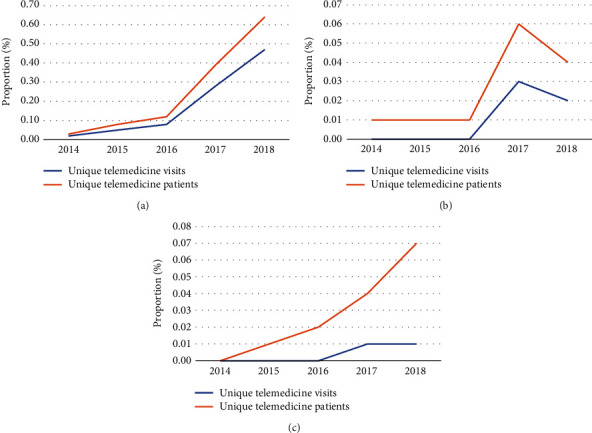
Trends in unique telemedicine visits/patients by encounter type: (a) office visit, nonspecialized physician; (b) office visit, specialized physician; and (c) visit with nonphysician “professional”.

In the 2014-2018 period of study, the proportion of unique telemedicine visits and patients increased across types of encounters (Figure 2). The proportions were the highest and rose the steadiest for nonspecialized physicians (0.02% of visits to 0.47% of visits; 0.03% to 0.64% of patients) (Figure 2(a)). Telemedicine utilization rates were lower and more variable in their growth among specialized physician (0% of visits to 0.02% of visits; 0.01% to 0.04% of patients) (Figure 2(b)) and nonphysician “professional” (0% of visits to 0.01% of visits; 0% to 0.07% of patients) (Figure 2(c)) visits/patients.

**Figure 3:**
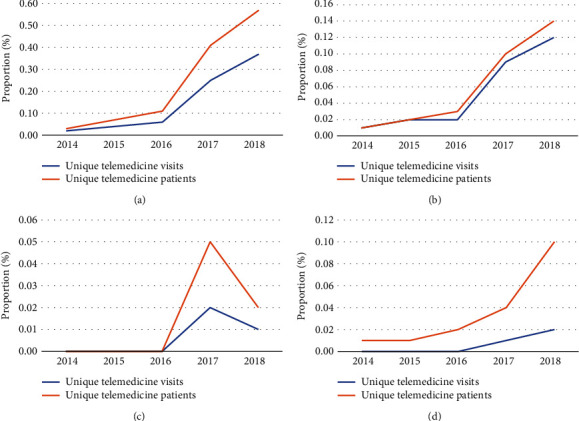
Trends in unique telemedicine visits/patients by provider type: (a) primary care, (b) urgent care, (c) orthopedic surgeon, and (d) other.

Upon stratifying telemedicine utilization by the type of provider (Figure 3), growth in the telemedicine proportions was observed from 2014 to 2018. Of the types of providers assessed, telemedicine made up the greatest proportion of primary care visits/patients (0.02% to 0.37% of visits; 0.03% to 0.57% of patients) (Figure 3(a)) and the least proportion of orthopedic surgeon visits/patients (0% to 0.01% of visits; 0% to 0.02% of patients) (Figure 3(c)). Telemedicine utilization rates steadily increased as a proportion of total visits/patients for primary care providers (Figure 3(a)), urgent care providers (0.01% to 0.12% of visits; 0.01% to 0.14% of patients) (Figure 3(b)), and “other” providers (0% to 0.02% of visits; 0.01% to 0.10% of patients) (Figure 3(d)). Interestingly, the proportions of unique telemedicine visits/patients showed a marked rise from 2016 to 2017, only to then drop, though not all the way to baseline rates, in 2018 (Figure 3(c)).

**Figure 4:**
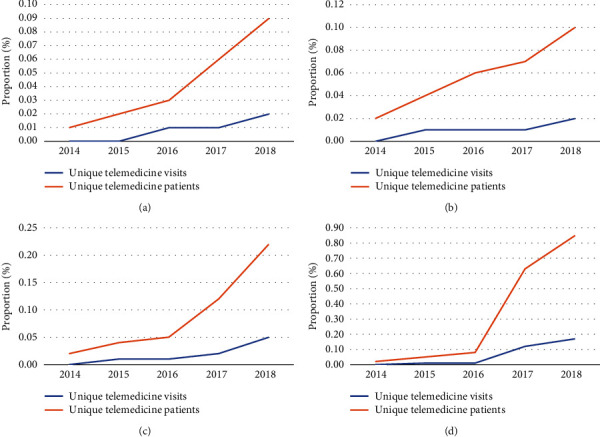
Trends in unique telemedicine visits/patients by region: (a) northeast, (b) north central, (c) south, and (d) west.

Telemedicine utilization increases from 2014 to 2018 were observed across all regions of study (Figure 4). Baseline rates of utilization in 2014 were comparably low across the United States, and the proportions of unique telemedicine visits/patients increased steadily across all of the regions. By 2018, the last year of study, the west region (0% to 0.17% of visits; 0.02% to 0.85% of patients) (Figure 4(d)) had the largest proportion of telemedicine utilization, followed by the south region (0% to 0.05% of visits; 0.02% to 0.22% of patients) (Figure 4(c)), with the northeast (0% to 0.02% of visits; 0.01% to 0.09% of patients) (Figure 4(a)) and north central (0% to 0.02% of visits; 0.02% to 0.10% of patients) (Figure 4(b)) regions demonstrating comparably low utilization.

Telemedicine utilization varied significantly across a range of telemedicine visit characteristics (Table 1). It was more likely to be used for musculoskeletal aftercare (OR 1.47, CI 1.34-1.61) than for low back/neck pain and less likely to be used for hip/knee pain (OR 0.75, CI 0.72-0.77) or other visits (OR 0.85, CI 0.83-0.87). Visits were less likely to be office visits with a specialized physician (OR 0.15, CI 0.14-0.15), visits with nonphysician “professional” (OR 0.03, CI 0.03-0.03), or other (OR 0.01, CI 0.01-0.01) than office visits with a nonspecialized physician. Similarly, visits were less likely to be with urgent care (OR 0.57, CI 0.55-0.59), with an orthopedic surgeon (OR 0.36, CI 0.34-0.39), or other (OR 0.51, CI 0.49-0.53) than with primary care. Telemedicine visits were significantly less likely to have a copayment (OR 0.27, CI 0.26-0.28). Finally, telemedicine visit data showed that there was an increasing likelihood of having a telemedicine visit from 2014 through 2018 (OR 18.3, CI 17.5-19.2).

Significant differences in utilization were also seen for several patient characteristics (Table 1). Females were more likely than males to have had a telemedicine visit (OR 0.86, CI 0.85-0.88). There was a decreasing likelihood of having a telemedicine visit for individuals < 55 years of age (OR 0.69, CI 0.67-0.71), increased Charlson comorbidity burden (OR 0.86, CI 0.84-0.88) to >2 (OR 0.68, CI 0.66-0.70), and an active opioid prescription (OR 0.90, CI 0.88-0.93). Patients in urban areas were more likely to have had a telemedicine visit than those in rural areas (OR 2.92, CI 2.79-3.05). In terms of location of residence, those that lived in the west region were the most likely to have had a telemedicine visit (OR 5.58, CI 5.36-5.81), followed by those from the south region (OR 2.28, CI 2.19-2.38), the north central region (OR 1.35, CI 1.28-1.42), and the northeast region (reference). Finally, patients with a median household income of <$45,000 were less likely (OR 0.74, CI 0.70-0.78) than those with a median household income of $45,000-$60,000 to have a telemedicine visit, and those with a median household income >$60,000 were more likely (OR 2.57, CI 2.49-2.65)

## 4. Discussion

Generally, there were very low rates of telemedicine utilization for musculoskeletal visits in the years preceding the COVID-19 pandemic. From 2014 to 2018, 0.020% of visits classified as musculoskeletal were conducted via telemedicine with 0.098% of patients having at least one visit via telemedicine. A stepwise increase was observed year-over-year, indicating an upward trend in the usage of telemedicine. The upward trends were similar across musculoskeletal visit subtype, with the exception of musculoskeletal aftercare which saw a slight decrease in unique telemedicine visits and patients in 2018. There was a rapid increase in the proportion of unique telemedicine patients, but the number of telemedicine visits per patient overall did not vary dramatically from year to year and remained around 1.13 (range 1.11-1.16). This finding indicates that telemedicine was becoming widely used and that increased overall telemedicine visits was not due to the same telemedicine patients having more frequent visits. This is in contrast to the literature as providers prefer using telemedicine for follow-up visits [18], a class of visit that typically outnumbers those such as consults or preoperative planning.

As evidenced by the low prepandemic rates of telemedicine, musculoskeletal care was virtually naïve to telemedicine. This is an important finding as it means the COVID-19 pandemic required the musculoskeletal field to drastically roll-out and adapt to telemedicine. Immature telemedicine programs were likely deployed, with equity a minimal focus in the era of crisis. With this low rate of prepandemic utilization established, characterizing the baseline of what types of visits were telemedicine and which patients participated in them is important for tracking how these trends evolved across the pandemic to the present day. These analyses are crucial for refining telemedicine programs and ensuring equity.

There were notable differences observed in telemedicine utilization across visit characteristics. Musculoskeletal aftercare yielded a higher proportion of telemedicine visits than low back/neck pain, a trend that will likely be found to be reversed during the COVID-19 pandemic due to a pause on elective musculoskeletal surgeries and a predictive increase in low back/neck pain as a result of more sedentary activity throughout the pandemic. Additionally, orthopedic surgeons previously utilized telemedicine at lower rates than primary care providers. Figures 2 and 3 together illustrate that the upward trends in telemedicine utilization were not as consistent for musculoskeletal visits as they were in primary care settings.

Interestingly, the regions with the highest proportion of unique patients utilizing telemedicine were the south (0.22% in 2018) and the west (0.85% in 2018), while the proportion in the northeast was only 0.09% in 2018. This data provides interesting prepandemic context, as institutions in the “hot spot” northeast and south regions of the United States were more likely to offer telemedicine at the onset of the pandemic [15]. While comparable claims data from 2019 to 2020 would provide additional insight into how the rate of telemedicine utilization has changed, the increase in telemedicine in these regions may have been the result of COVID-19 rather than simply a reflection of previous trends.

Unfortunately, at this time, telemedicine has not been adopted equally across the entire patient population. Here, female patients and older patients were more likely to have had a telemedicine visit. Conversely, patients that have more comorbidities, have lower incomes, and live in rural areas are less likely to have had a telemedicine visit. These groups already suffer from health disparities—disparities that translate into prepandemic telemedicine utilization, as well.

The observed disparities could be due to a variety of factors. For instance, patients with lower incomes may have more limited access to necessary telemedicine technology and may therefore be less likely to have had a telemedicine visit. This disparity is especially damaging as individuals with lower income could theoretically benefit the most from having flexibility in the location in which they attend their appointments. Previous studies have also shown that individuals with lower socioeconomic status are more likely to have a failed telemedicine video visit [19, 20]. Flexibility in the modality of telemedicine visit may help address this disparity, as telephone visits have been shown to be comparably successful across income groups [19]. Additionally, those living in urban areas have increased access to musculoskeletal care nearby; therefore, one would expect rural areas to have higher rates of telemedicine utilization. However, this is not the case in our data, as telemedicine utilization rates are higher for those from urban areas. Urban clinics may have more resources to develop and implement telemedicine programs, resulting in a roll-out effect that favors their earlier adoption of telemedicine practices. It is clear that significant disparities existed in telemedicine preceding COVID-19, many of which may have been exacerbated by the pandemic. Prepandemic telemedicine usage must be known to accurately assess and address the impact of COVID-19 on these disparities.

With regard to assessment of the trends of annual telemedicine utilization, a sharp uptick is observed in 2016. Several policy factors, germane to the period leading into 2016, might be implicated in the observed uptick. First, health plans began to expand the coverage of telemedicine services. The Center for Medicare & Medicaid Services (CMS) reported that, from 2014 to 2016, there was a 48.3% increase in the number of plans that covered telemedicine services [21]. Additionally, states began to increasingly regulate private insurers to cover telemedicine services [22]. This expansion of the coverage of telemedicine services by health plans likely resulted in the subsequent increase in telemedicine visits. Another potential policy driver of telemedicine involves the advent of Accountable Care Organizations (ACOs). Many ACOs sought to save costs through bundled payment reimbursement schemes, charging a single price up front for all associated costs of a procedure. In an effort to improve cost-savings, it is possible that ACOs turned to telemedicine as a cheaper modality of patient care in musculoskeletal settings.

### 4.1. Limitations

This study is limited in its use of commercial claims data as it omits many older and lower-income patients that are enrolled in Medicare and Medicaid, respectively. Additionally, the lack of information on telemedicine modality may limit this research, as the trends in telephone visits may differ from those of video visits; further stratification of telemedicine modality may provide additional insight into disparities in telemedicine.

## 5. Conclusions

This study demonstrates that there were overall low rates of telemedicine, particularly in musculoskeletal care, in the prepandemic period, though utilization trended upward from 2014 to 2018. Those with increased comorbidities, lower incomes, and living in rural areas had lower rates of telemedicine utilization in the prepandemic period. This information provides much needed information on previous trends in telemedicine, and this baseline can be used for comparison as research is conducted on telemedicine in the era of COVID-19.

## Figures and Tables

**Table 1:** Telemedicine use by study variables; absolute (unadjusted) numbers on the left and adjusted odds ratios with 95% confidence intervals on the right.

	Telemedicine utilization				
	No	Yes	%	OR	95% CI
Telemedicine visit characteristics					
*Diagnosis*					
Low back/neck pain	62,521,546	14,573	0.02	Ref	—
Hip/knee pain	12,521,659	3618	0.03	0.75^∗^	0.72-0.77
Musculoskeletal aftercare	3,320,970	517	0.02	1.47^∗^	1.34-1.61
Other	111,897,399	18,964	0.02	0.85^∗^	0.83-0.87
*Encounter type*					
Office visit; nonspecialized physician	19,864,435	29,867	0.15	Ref	—
Office visit; specialized physician	31,157,145	3574	0.01	0.15^∗^	0.14-0.15
Visit with nonphysician “professional”	91,163,682	3692	0	0.03^∗^	0.03-0.03
Other	48,076,312	539	0	0.01^∗^	0.01-0.01
*Provider type*					
Primary care	21,402,964	24,407	0.11	Ref	—
Urgent care	7,338,940	3389	0.05	0.57^∗^	0.55-0.59
Orthopedic surgeon	19,486,716	1272	0.01	0.36^∗^	0.34-0.39
Other	142,032,954	8604	0.01	0.51^∗^	0.49-0.53
*Copayment*					
Yes	65,571,904	8391	0.01	0.27^∗^	0.26-0.28
*Year of visit*					
2014	55,513,807	2099	0	Ref	—
2015	33,998,374	2504	0.01	2.1^∗^	1.98-2.22
2016	35,029,043	3698	0.01	3.21^∗^	3.04-3.39
2017	32,745,728	11,524	0.04	12.1^∗^	11.6-12.7
2018	32,974,622	17,847	0.05	18.3^∗^	17.5-19.2
Patient characteristics					
*Sex*					
Male	75,401,273	17,573	0.02	Ref	—
Female	114,860,301	20,099	0.02	0.86^∗^	0.85-0.88
*Age*					
<55 years	124,306,442	28,406	0.02	Ref	—
≥55 years	65,955,132	9266	0.01	0.69^∗^	0.67-0.71
*Charlson comorbidity burden*					
0	103,308,569	23,356	0.02	Ref	—
1	43,267,583	8392	0.02	0.86^∗^	0.84-0.88
≥2	43,685,422	5924	0.01	0.68^∗^	0.66-0.70
*Active opioid prescription*	16,915,529	4756	0.03	0.9^∗^	0.88-0.93
*Residence rurality*					
Urban	158,354,337	34,565	0.1	2.92^∗^	2.79-3.05
Rural	22,780,429	2341	0.1	Ref	—
*Residence geographic region*					
Northeast	39,662,302	2825	0.01	Ref	—
North central	40,733,490	4149	0.01	1.35^∗^	1.28-1.42
South	73,553,316	12,705	0.02	2.28^∗^	2.19-2.38
West	34,737,212	17,907	0.05	5.58^∗^	5.36-5.81
Unknown	1,575,254	86	0.01	5.24^∗^	4.19-6.54
*Median household income*					
<$45,000	12,106,955	1477	0.01	0.74^∗^	0.70-0.78
$45,000–$60,000	75,903,787	11,760	0.02	Ref	—
>$60,000	18,873,830	7608	0.04	2.57^∗^	2.49-2.65
Unknown	83,377,002	16,827	0.02	1.76^∗^	1.72-1.81

^∗^ *p* < 0.001.

## Data Availability

This study utilized the Truven MarketScan database, a commercial dataset produced by Truven Health Analytics (copyright © 2017 Truven Health Analytics Inc.).
